# Ovarian granulosa cell tumor and clomiphene citrate resistance. A case report and review of the literature

**DOI:** 10.5935/1518-0557.20180056

**Published:** 2018

**Authors:** Olga Triantafyllidou, George Sigalos, Irida Oikonomou, Nikos Vlahos

**Affiliations:** 1 Reproductive Medicine Unit, "Leto" Maternity Hospital, Athens, Greece; 2 Medical school, University of Athens, Greece; 3 2_nd_ Department of Obstetrics and Gynecology, "Aretaieion" Hospital, University of Athens, Greece

**Keywords:** granulosa cell tumor, sex-cord ovarian tumor, clomiphene resistance, infertility

## Abstract

Granulosa cell tumors (GCTs) account for less than 5% of all ovarian malignancies, occur in younger ages, are usually diagnosed in their early stages, and have a good prognosis. GCTs usually present with features of hyperestrogenism. This paper reports the unusual case of an adult with a GCT with manifestations including amenorrhea, mild hirsutism, infertility, clomiphene citrate (CC) resistance (CC), mildly elevated testosterone, high anti-Müllerian hormone (AMH) and normal estrogen levels. The patient was initially diagnosed with polycystic ovarian syndrome (PCOS), and after four attempts at ovarian stimulation she was diagnosed with CC resistance. The patient later underwent laparoscopic evaluation on account of a solid mass on her left ovary. The pathology report described it as a borderline adult type GCT and four weeks after surgery she had a positive pregnancy test. Twelve months after delivery, the patient had no obvious symptoms of disease and her menstrual cycle was normal. Serial measurements of serum inhibin B, AMH, estrogen, and testosterone levels were within normal range. In conclusion, the resistance to clomiphene manifested by the patient might be explained by a potential mechanism implicating inhibin B and AMH due to the presence of a GCT. Further studies are required to evaluate the role of AMH and Inhibin B in the mechanism of CC resistance in women with PCOS.

## INTRODUCTION

Granulosa cell tumors (GCTs) constitute more than 70% of sex cord-stromal tumors, but account for less than 5% of all ovarian malignancies (Pectasides *et al.,* 2008; Ugianskiene *et al.,* 2014). GCTs appear in two different histological types: adult (AGCT) and juvenile (JGCT), which differ in histopathology, clinical presentation, and prognosis. AGCTs are far more common, accounting for 95% of all GCTs; they are usually diagnosed in perimenopausal and postmenopausal women aged 50-55 years. JGCTs are rare tumors, representing only 5% of all GCTs, and affect mostly premenarcheal girls and young women (Ugianskiene *et al.,* 2014; Bryk *et al.,* 2015). When compared to epithelial ovarian tumors, GCTs occur in younger individuals, are usually diagnosed in earlier stages, and offer a significantly better prognosis with long-term disease-free survival rates of up to 90% (Schumer & Cannistra, 2003). According to recent data from Surveillance, Epidemiology and End Results (SEER), nearly 57% of GCTs are stage I tumors and may therefore be managed surgically, with the intent of completely resecting the tumor (Schumer & Cannistra, 2003; Piura *et al.,* 1994; Kottarathil *et al.,* 2013).

GCTs usually present with features of hyperestrogenism. Therefore, symptoms usually revolve around altered menstrual patterns (32.8%) including menorrhagia, metrorrhagia or irregular vaginal bleeding (Ranganath *et al*., 2008; Koukourakis *et al.,* 2008). Postmenopausal bleeding is the most common finding in the postmenopausal age group; association with endometrial hyperplasia occurs in 25-50% of the cases (Schumer & Cannistra, 2003).

This report described an unusual case of AGCT presenting with amenorrhea, infertility, clomiphene citrate (CC) resistance, and normal estrogen levels.

## CASE REPORT

A 32-year-old Caucasian woman came to our service with complaints of amenorrhea and infertility. She had a history of two previous failed attempts at ovarian stimulation with CC. She had been successfully treated for amenorrhea with progestin (norethisterone 5 mg/day, Primolut, Bayer, Germany) for 10 days in a monthly fashion.

Clinical examination revealed mild hirsutism (upper lip, chin, and upper abdominal area) without other signs of virilization (she was not assigned a Ferriman-Gallwey score due to extensive use of laser for hair removal). Pelvic examination did not reveal clitoromegaly. Her uterus and adnexa were of normal size. Transvaginal ultrasound evaluation confirmed the above findings; no signs of polycystic morphology were seen in her ovaries. A uterine septum was suggested and confirmed by hysterosalpingography (HSG). Her partner’s semen parameters were normal. Hormonal analysis on day 3 of her menstrual cycle showed the following: estradiol 38pg/ml; FSH 3.6 IU/l; testosterone 68ng/dl (normal range 5-52ng/dl); LH 22.8 IU/l; and Anti-Müllerian hormone (AMH) 179pmol/l. A possible diagnosis of polycystic ovarian syndrome (PCOS) based on the Rotterdam criteria was considered (Rotterdam ESHRE/ASRM-Sponsored PCOS Consensus Workshop Group, 2004).

After hysteroscopic resection of the septum, the patient proceeded with two additional ovarian stimulation cycles with clomiphene citrate 100 mg/day (Clomiphene citrate, Anfarm Hellas, Greece) for 5 days (from day 3 to day 7) with no response. Ultrasound examination performed after the last attempt indicated her left ovary was mildly enlarged possibly because of a solid mass. Contrast-enhanced magnetic resonance imaging (MRI) scans confirmed the presence of an irregular, solid mass with a diameter of 36 mm within the substance of the left ovary without additional findings. Tumor markers (CA125: 14.7 U/ml, CEA: 1.5 ng/ml, αFP: 4.0 ng/ml) were within normal range.

Laparoscopic examination showed her left ovary was enlarged, with no obvious surface anomalies. After peritoneal washing cytology, her ovary was bivalved to reveal a well-defined solid mass that was easily separated from the surrounding ovarian tissue. The surface of the tumor was yellowish and friable. The tumor was removed and contained in an endobag. Her right ovary and both fallopian tubes were normal. No other signs of disease were noted in the peritoneal cavity and the procedure was completed.

The pathology report described a borderline adult GCT. Staining by immunohistochemistry was positive for inhibin and partly positive for calherin. Nearly 10% of the cells stained positive for proliferation marker Κi-67. Examination with a microscope showed 0-2 mitoses per 10 high power field. Inhibin B serum levels measured upon histological confirmation two weeks after the procedure were within normal range (20pg/ml).

The patient was informed of the pathology results and was scheduled for surgical staging. On the day of the procedure, the patient had a positive pregnancy test confirmed by serum β-hCG, and the procedure was cancelled. A singleton viable pregnancy was confirmed by ultrasound examination two weeks later. The patient was advised to proceed with surgical staging, with a tentative date for the procedures somewhere around the 12^th^ week of gestation. The risks and benefits were explained to her, but she refused to carry on with it. During her pregnancy she had serial ultrasound images of her ovaries taken along with measurements of inhibin B serum levels, which remained within normal levels. The patient underwent a planned cesarean section under epidural anesthesia at 40 weeks of gestation. During the procedure there was no macroscopic evidence of recurrent disease on her left ovary or anywhere else in the peritoneal cavity. Complete surgical staging, including peritoneal washings, left adnexectomy, exploration of peritoneal cavity, multiple peritoneal biopsies, and omentectomy were performed. Cytology and histology tests were negative for disease.

Twelve months after delivery the patient had no obvious symptoms of disease and her menstrual cycle was normal. Serial measurements of serum inhibin B, AMH, estrogen, and testosterone levels were within normal range.

## DISCUSSION

Granulosa cell tumors (GCTs) originate from somatic cells of the sex cords of the ovary. Major functions of the granulosa cells include production of sex steroids and various other peptides required for folliculogenesis and ovulation (Kottarathil *et al.*, 2013). Ovarian GCTs are categorized as sex cord-stromal tumors and behave as borderline malignant tumors (Pectasides *et al.*, 2008; Fox *et al.*, 1975). The 10-year survival rate of patients with early stage (I or II) disease is excellent (up to 90%), while prognosis is less favorable for women with late stage disease, with 10-year survival rates at 50-65% for stage II disease and 17-33% for disease stages III and IV (Schumer & Cannistra, 2003). Late recurrence - 20 to 30 years after initial surgery - is one of the characteristic features of GCTs (Zhang *et al.*, 2007).

GCTs are often hormonally active tumors and may secret estrogen, inhibin (especially the beta subunit), and AMH, since these are normally produced by granulosa cells. Inhibin is produced in small preantral and antral follicles (Danforth *et al.*, 1998). Lappöhn *et al.* (1989) first demonstrated the efficacy of inhibin B as a marker for both primary and recurrent disease and showed that increased inhibin B levels may predate clinical recurrence by up to 20 months. Following complete surgical removal of the tumor, inhibin levels are expected to fall to normal range within one week (Koukourakis *et al.*, 2008). Elevated serum AMH levels are also highly specific for GCTs (Koukourakis *et al.*, 2008). AMH - a product of granulosa cells in the preantral and small antral follicles - inhibits primordial follicle recruitment and the responsiveness of growing follicles to Follicle-Stimulating Hormone (FSH) (Zahid, 2014). AMH may also predate clinical recurrence by up to 11 months (Koukourakis *et al.*, 2008). Several studies described AMH as a reliable tumor marker for either diagnosis or recurrent disease, with sensitivities ranging between 76% and 100% (Long *et al*., 2000; Chang *et al.*, 2009). Further studies are required to define which tumor markers are more reliable to detect and manage GCTs. In rare cases of androgen secreting GCT, as in our case (testosterone levels were slightly elevated, 68ng/dl), patients may present with symptoms mimicking PCOS syndrome such as hirsutism and amenorrhea.

GCTs usually present as large unilateral masses (Ranganath *et al*., 2008). Radiological criteria suggestive of malignant ovarian masses include thick, irregular walls, and septa; papillary projections and solid echogenic foci (Jeong *et al.*, 2000). An enlarged uterus with a thickened endometrium is often seen on account of the effect of estrogen. The incidence of peritoneal spread is low and tumors are rarely bilateral. Symptoms are usually nonspecific and include abdominal pain (41.1%) and distension (26.4%) or are associated with hyperestrogenism (menorrhagia, irregular menstrual bleeding) (Ranganath *et al*., 2008). Infertility may occur secondary to high inhibin levels. GCTs occasionally have a Sertoli-Leydig cell component and produce androgens, which may cause virilizing symptoms (Koukourakis *et al.*, 2008).

Surgery remains as the method of choice to manage GCTs. The steps and principles of surgical staging are the same for all borderline ovarian tumors and include thorough exploration of the peritoneal cavity, cytology washings, multiple peritoneal biopsies, and omentectomy. Thrall *et al.* (2011) mentioned that secondary surgical staging is necessary in unstaged cases of GCT; in two of eight cases, patients were upstaged. After childbearing, patients with GCTs are advised to undergo total abdominal hysterectomy and bilateral salpingo-oophorectomy with optimal cytoreduction to eliminate any residual disease. Despite the lack of prospective randomized studies, fertility sparing surgery is recommended for young women. As the incidence of bilateral disease is low (2%) (Ranganath *et al*., 2008), performing a wedge biopsy on the contralateral ovary is controversial. Lymph node metastases are rarely observed in women with GCTs, therefore pelvic and para-aortic lymphadenectomy may not be included in surgical staging. Endometrial sampling is needed in women with menstrual bleeding, since up to 10% of the patients may present with endometrial hyperplasia or endometrial cancer (2%). Patients with early-stage GCTs (stages I and II) enjoy very good prognosis with a 5-year disease-free survival rate of 89%, and usually do not require postoperative treatment (Ranganath *et al*., 2008). Some authors suggested that patients with advanced-stage or recurrent disease might benefit from postoperative chemotherapy (Park *et al*., 2012; Uygun *et al.*, 2003), but other studies found no association between the prescription of chemotherapy and improved survival rates (Al-Badawi *et al.*, 2002; Chan *et al.*, 2005).

Late recurrence occurs in a fraction of GCTs. In a study enrolling 93 patients, Park *et al*. (2012) reported that none of the patients with early stage disease submitted to optimal debulking had tumor recurrence. On the other hand, Wilson *et al*. (2015) found recurrent disease in 32% (51/160) of the cases in a large retrospective series of 160 FIGO stage I AGCTs. The median time to relapse was 12 years, underscoring the typical biological behavior of AGCTs with late relapse. Due to the rarity of AGCTs, treatment options for recurrent disease have not been standardized.

This report discussed the case of a patient presented with amenorrhea and infertility. She was diagnosed with a GCT and successfully treated with surgery. Her tumor was staged as Ia disease at the time of initial surgery. Therefore, during her cesarean section she underwent fertility preserving surgery with ipsilateral salpingo-oophorectomy. Surgical staging was based on peritoneal washing cytology, multiple peritoneal biopsies, and omentectomy. The symptoms manifested by the patient - amenorrhea and high testosterone level - were consistent with PCOS according to the Rotterdam criteria. Initial hormonal evaluation revealed a low level of FSH (3.6 IU/l) with normal E2 level (38pg/ml), normal AFC (10) with elevated LH levels (22.8 IU/l), mildly elevated testosterone (68ng/dl), and very high AMH (179pmol/l) levels, all of which consistent with her initial diagnosis of PCOS.

Since clomiphene citrate (CC) is the treatment of choice to induce ovulation in women with PCOS, the patient underwent four ovarian stimulation cycles with 100 mg/day of CC for 5 days (from day 3 to day 7) with no response, and was diagnosed with CC resistant PCOS. Clomiphene resistance - defined as failure to ovulate after receiving CC for 5 days per cycle, for at least three cycles - is a common event and occurs in approximately 15-40% of the women with PCOS (Saha *et al*., 2013).

The patient was not suspected for GCT, and therefore her inhibin B levels were not measured. Moreover, her estradiol levels were in the low-to-normal range and never increased during stimulation cycles. When the diagnosis was confirmed (two weeks after laparoscopy), her inhibin B levels were within normal range (20pg/ml). Her AMH levels were elevated, but in the absence of other findings the increase was attributed to PCOS. It is unclear why the patient had such clinical presentation and her estradiol levels were low. Retrospectively, we suspected that her low FSH levels might be explained by possible increases in inhibin B levels before surgery, since increases in inhibin B lead to declines in FSH levels (Al-Badawi *et al*., 2002).

The fact that her ovarian function resumed immediately after the removal of the tumor and that she was able to ovulate and become pregnant, in addition to the fact that her menstrual cycles were completely normal after delivery, suggest a potential mechanism implicating inhibin B or even AMH in clomiphene resistance.

In conclusion, this report presented the case of a patient with infertility, anovulation, and clomiphene resistance caused by a GCT without hyperestrogenism treated successfully with surgery. Further studies are required to assess the role of AMH and Inhibin B in the mechanism of clomiphene citrate resistance in women with PCOS.

## References

[r1] Al-Badawi IA, Brasher PM, Ghatage P, Nation JG, Schepansky A, Stuart GC (2002). Postoperative chemotherapy in advanced ovarian granulosa cell
tumors. Int J Gynecol Cancer.

[r2] Bryk S, Färkkilä A, Bützow R, Leminen A, Heikinheimo M, Anttonen M, Riska A, Unkila-Kallio L (2015). Clinical characteristics and survival of patients with an
adult-type ovarian granulosa cell tumor: a 56-year single-center
experience. Int J Gynecol Cancer.

[r3] Chan JK, Zhang M, Kaleb V, Loizzi V, Benjamin J, Vasilev S, Osann K, Disaia PJ (2005). Prognostic factors responsible for survival in sex cord stromal
tumors of the ovary--a multivariate analysis. Gynecol Oncol.

[r4] Chang HL, Pahlavan N, Halpern EF, MacLaughlin DT (2009). Serum Müllerian Inhibiting Substance/anti-Müllerian
hormone levels in patients with adult granulosa cell tumors directly
correlate with aggregate tumor mass as determined by pathology or
radiology. Gynecol Oncol.

[r5] Danforth DR, Arbogast LK, Mroueh J, Kim MH, Kennard EA, Seifer DB, Friedman CI (1998). Dimeric inhibin: a direct marker of ovarian aging. Fertil Steril.

[r6] Fox H, Agrawal K, Langley FA (1975). A clinicopathologic study of 92 cases of granulosa cell tumor of
the ovary with special reference to the factors influencing
prognosis. Cancer.

[r7] Jeong YY, Outwater EK, Kang HK (2000). Imaging evaluation of ovarian masses. Radiographics.

[r8] Kottarathil VD, Antony MA, Nair IR, Pavithran K (2013). Recent advances in granulosa cell tumor ovary: a
review. Indian J Surg Oncol.

[r9] Koukourakis GV, Kouloulias VE, Koukourakis MJ, Zacharias GA, Papadimitriou C, Mystakidou K (2008). Pistevou-Gompaki K, Kouvaris J, Gouliamos A. Granulosa cell tumor
of the ovary: tumor review. Integr Cancer Ther.

[r10] Lappöhn RE, Burger HG, Bouma J, Bangah M, Krans M, de Bruijn HW (1989). Inhibin as a marker for granulosa-cell tumors. N Engl J Med.

[r11] Long WQ, Ranchin V, Pautier P, Belville C, Denizot P, Cailla H, Lhommé C, Picard JY, Bidart JM, Rey R (2000). Detection of minimal levels of serum anti-Müllerian
hormone during follow-up of patients with ovarian granulosa cell tumor by
means of a highly sensitive enzyme-linked immunosorbent
assay. J Clin Endocrinol Metab.

[r12] Park JY, Jin KL, Kim DY, Kim JH, Kim YM, Kim KR, Kim YT, Nam JH (2012). Surgical staging and adjuvant chemotherapy in the management of
patients with adult granulosa cell tumors of the ovary. Gynecol Oncol.

[r13] Pectasides D, Pectasides E, Psyrri A (2008). Granulosa cell tumor of the ovary. Cancer Treat Rev.

[r14] Piura B, Nemet D, Yanai-Inbar I, Cohen Y, Glezerman M (1994). Granulosa cell tumor of the ovary: a study of 18
cases. J Surg Oncol.

[r15] Ranganath R, Sridevi V, Shirley SS, Shantha V (2008). Clinical and pathologic prognostic factors in adult granulose
cell tumors of the ovary. Int J Gynecol Cancer.

[r16] Rotterdam ESHRE/ASRM-Sponsored PCOS Consensus Workshop
Group (2004). Revised 2003 consensus on diagnostic criteria and long-term
health risks related to polycystic ovary syndrome. Fertil Steril.

[r17] Saha L, Kaur S, Saha PK (2013). N-acetyl cysteine in clomiphene citrate resistant polycystic
ovary syndrome: A review of reported outcomes. J Pharmacol Pharmacother.

[r18] Schumer ST, Cannistra SA (2003). Granulosa cell tumor of the ovary. J Clin Oncol.

[r19] Thrall MM, Paley P, Pizer E, Garcia R, Goff BA (2011). Patterns of spread and recurrence of sex cord-stromal tumors of
the ovary. Gynecol Oncol.

[r20] Ugianskiene A, Grove A, Soegaard-Andersen E (2014). Adult granulosa cell tumor of the ovary: a retrospective study of
37 cases. Eur J Gynaecol Oncol.

[r21] Uygun K, Aydiner A, Saip P, Kocak Z, Basaran M, Dincer M, Topuz E (2003). Clinical parameters and treatment results in recurrent granulosa
cell tumor of the ovary. Gynecol Oncol.

[r22] Wilson MK, Fong P, Mesnage S, Chrystal K, Shelling A, Payne K, Mackay H, Wang L, Laframboise S, Rouzbahman M, Levin W, Oza AM (2015). Stage I granulosa cell tumours: A management conundrum? Results
of long-term follow up. Gynecol Oncol.

[r23] Zahid N (2014). Role of Anti-Mullerian Hormone (AMH) in Polycystic Ovary Syndrome
(PCOS)? A Mini Review. Reprod Syst Sex Disord.

[r24] Zhang M, Cheung MK, Shin JY, Kapp DS, Husain A, Teng NN, Berek JS, Osann K, Chan JK (2007). Prognostic factors responsible for survival in sex cord stromal
tumors of the ovary--an analysis of 376 women. Gynecol Oncol.

